# A mediating effect of sense of coherence on the association between work-family conflict and workplace ostracism among medical staff

**DOI:** 10.3389/fpsyt.2025.1584004

**Published:** 2026-02-02

**Authors:** Na Li, Huifeng Zhang, Hui Zhao, Congmin Zhang, Huinan Zhang, Fenghui Ma, Xiaojia Tang, Cuicui Wang, Jing Huang

**Affiliations:** 1 Department of Health and Management, Tangdu Hospital, Fourth Force Military Medical University, Xi’an, China; 2 Department of Health Examination Center, The Second Affiliated Hospital, Dalian Medical University, Dalian, China; 3 Henan Vocational College of Nursing, Anyang, China; 4 Department of Rehabilitation Medicine, Northern Jiangsu People’s Hospital, Yang Zhou, China; 5 Department of Gastroenterology, 986th Hospital Affiliated to Fourth Force Medical University, Xi’an, China

**Keywords:** mental health, medical staff, workplace ostracism, work-family conflict, sense of coherence, mediation effect

## Abstract

**Background:**

The mental health of medical professionals has garnered increasing attention in recent years. This study aims to explore the relationships and interactions among workplace ostracism, work-family conflict, and sense of coherence (SOC) within this population.

**Methods:**

From January 2022 to December 2023, a survey was conducted involving 527 medical staff from three diverse hospitals. The research utilized the Work-Family Conflict Scale, the Workplace Ostracism Scale, and the Sense of Coherence Scale. Descriptive statistics and correlation analyses were performed on the collected data. Additionally, mediation analysis was employed to assess indirect effects, utilizing bootstrap sampling to estimate confidence intervals for these mediated effects. Simple slope analysis was also conducted to interpret significant interaction effects within moderation models.

**Results:**

A significant positive correlation was found between work-family conflict and workplace ostracism (correlation coefficient = 0.613; P < 0.001). Furthermore, sense of coherence partially mediates the relationship between work-family conflict and workplace ostracism (β = 0.330; P < 0.001). As levels of sense of coherence increase, the impact of work-family conflict on workplace ostracism diminishes progressively.

**Conclusion:**

Work-family conflict is a significant positive predictor of workplace ostracism among medical staff; moreover, sense of coherence serves as a mediator in this relationship—an effect that is particularly pronounced among those with lower levels of sense of coherence.

## 1 Introduction

Mental health issues have received growing global attention in recent years (1, 2). Among key groups responsible for safeguarding public health, medical personnel are particularly vulnerable due to substantial work-related pressures and diverse psychological challenges (3). Consequently, their mental well-being demands immediate and sustained attention. There is also increasing recognition that the mental health of medical staff constitutes a foundational element of high-quality and sustainable healthcare systems (4, 5).

Among the various factors influencing the mental health of medical professionals, three indicators have emerged as critical in reflecting the psychological status of occupational populations: workplace ostracism, work-family conflict, and Sense of Coherence (SOC) (6–8). Empirical evidence has further demonstrated that these three factors significantly affect the mental well-being of healthcare workers (9–11).

Workplace ostracism refers to the experience in which an individual or group is excluded, ignored, or socially disregarded by others in the work environment. This phenomenon can impair the development and maintenance of positive interpersonal relationships, hinder career progression, and damage professional reputation (12). Notably, although workplace ostracism is prevalent within the healthcare sector, it often remains invisible—yet it may still negatively impact patient care quality (11, 13). For medical professionals, the consequences extend beyond feelings of isolation: workplace ostracism substantially reduces job satisfaction and serves as a key determinant of stress levels and perceived psychological well-being (11).

In addition to workplace ostracism, work-family conflict represents another significant threat to the mental health of medical staff. Defined as a form of inter-role conflict, it occurs when the demands of work and family roles are incompatible, making it difficult for individuals to meet responsibilities in both domains simultaneously (14). It manifests in three primary forms: time-based, strain-based, and behavior-based conflict. A survey of 1,540 nurses confirmed this association, revealing a significant positive correlation between work-family conflict and burnout (15).

In contrast to these negative influences, SOC functions as a protective psychological resource. It reflects an individual’s ability to manage stress through perceiving life as comprehensible, manageable, and meaningful (16). A strong SOC is positively associated with better mental health outcomes, as it enables individuals to interpret challenges as opportunities for personal growth (17). Supporting this perspective, a systematic review found that higher SOC levels among nurses are negatively associated with burnout, depression, anxiety, and job dissatisfaction (18).

Despite robust evidence linking workplace ostracism, work-family conflict, and SOC individually to mental health outcomes, research on their interplay remains limited. The extent to which these factors jointly and interactively influence the mental well-being of medical personnel remains unclear. To address this critical gap, this study analyzes survey data from healthcare workers to achieve two primary objectives: (1) to examine the relationships and interactions among these three constructs, and (2) to identify associated risk factors. The findings aim to inform the development of targeted interventions.

## 2 Materials and methods​

### 2.1 Study design and setting​

This was a cross-sectional survey conducted between January 2022 and December 2023 among healthcare professionals from three tertiary general hospitals in China, including Tangdu Hospital (Fourth Military Medical University), the Second Affiliated Hospital of Dalian Medical University, and Northern Jiangsu People’s Hospital. The study aimed to explore the relationships and interactive effects between workplace ostracism, work-family conflict, sense of coherence (SOC), and mental health outcomes among medical staff. Data were collected via an online electronic questionnaire platform to ensure efficiency and standardization of data collection.

### 2.2 Study population and sampling​

This study was conducted in compliance with the *Declaration of Helsinki* and approved by the Ethics Committee of the Second Affiliated Hospital of Dalian Medical University (Approval No.: 01; Approval Date: April 25, 2021).

Convenience sampling was adopted to recruit eligible participants. The sample size was determined based on the requirement for multivariate analysis (at least 10 participants per variable) (22). Given the inclusion of 49 variables (predictors, mediator, outcome, and control variables), a target sample size of 490 was set.

Inclusion criteria: (1) Currently employed healthcare professionals, including physicians, nurses, and medical technicians; (2) Aged 18–60 years.

Exclusion criteria: (1) Retirees; (2) Individuals on leave for more than 6 months; (3) Questionnaires completed in an unreasonably short time (defined as < 3 minutes, based on the average completion time of pre-survey); (4) Responses with contradictory information.

### 2.3 Data collection procedures​

Questionnaires were distributed through *Wen Juan Xing* (a validated electronic data capture platform in China; https://www.wjx.cn/). Prior to questionnaire administration, participants were provided with a detailed informed consent statement outlining the study purpose, procedures, voluntary participation, right to withdraw, and privacy protection measures. Since the survey was conducted online, participants’ completion and submission of the questionnaire were deemed as implicit consent, and no written signature was required. All items were mandatory, and each IP address was restricted to one submission to avoid duplicate responses.

A total of 550 questionnaires were distributed, and 23 were excluded due to invalid responses (e.g., contradictory answers, hasty completion). Finally, 527 valid questionnaires were included in the statistical analysis, yielding an effective recovery rate of 95.82%.

### 2.4 Measurement instruments​

Detailed information on the full questionnaire content is provided in the **Supplementary Material**.

#### 2.4.1 Sociodemographic Questionnaire​

A self-designed sociodemographic questionnaire was used to collect participants’ basic information, including gender, age, marital status, developmental stage of the youngest child (if applicable), educational level, professional title, job position, and average number of night shifts per week.

#### 2.4.2 Work-Family Conflict Scale (WFCS)

The WFCS developed by Carlson et al. (14) was adopted to measure work-family conflict. This scale consists of 18 items across three dimensions: behavioral-based conflict, strain-based conflict, and time-based conflict. Responses were rated on a 5-point Likert scale (1 = “strongly disagree” to 5 = “strongly agree”). Total scores range from 18 to 90, with higher scores indicating higher levels of work-family conflict. In the present study, the Cronbach’s α coefficient of the WFCS was 0.95, indicating excellent internal consistency.

#### 2.4.3 Workplace Ostracism Scale (WOS)

A unidimensional 10-item WOS (19) was used to assess perceived workplace ostracism. Responses were scored on a 5-point Likert scale (1 = “completely disagree” to 5 = “strongly agree”). Total scores range from 10 to 50, with higher scores reflecting greater perceived workplace ostracism. The Cronbach’s α coefficient of the WOS in this study was 0.88, indicating good internal consistency.

#### 2.4.4 Sense of Coherence Scale (SOC-13)

The Chinese version of the SOC-13, revised by Bao et al. (20), was employed to measure participants’ sense of coherence. This scale includes 13 items across three dimensions: comprehensibility, manageability, and meaningfulness. Responses were rated on a 7-point Likert scale (1 = “never” to 7 = “very often”), with 5 items reverse-scored. Total scores range from 13 to 91, with higher scores indicating a stronger sense of coherence. According to established criteria (21), scores of 13–63 indicate a low level of coherence, 64–79 indicate a moderate level, and 80–91 indicate a high level. The Cronbach’s α coefficient of the SOC-13 in the current study was 0.76, indicating acceptable internal consistency.

### 2.5 Statistical analysis​

All statistical analyses were performed using SPSS Statistics version 26.0 (IBM Corp., Armonk, NY, USA) and Stata version 16.0 (StataCorp LLC, College Station, TX, USA). Descriptive statistics were applied to characterize the study population and key variables: continuous variables (e.g., age, scale scores) were presented as mean ± standard deviation (M ± SD), while categorical variables (e.g., gender, marital status) were described by frequency and percentage (n, %). Pearson correlation analysis was conducted to examine bivariate linear relationships among continuous variables (work-family conflict, sense of coherence, workplace ostracism).

Multiple linear regression models were used to identify significant predictors of workplace ostracism, work-family conflict, and sense of coherence, with covariates (gender, age, education level, marital status, professional title, child stage, position, average number of night shifts per week) included in the adjusted models. For mediation analysis, the bootstrap sampling method (5,000 resamples) was employed to test the indirect effect and indirect effect (via sense of coherence) were estimated, with 95% confidence intervals (CIs) used to determine significance (non-overlapping with 0 indicated a significant effect).

For moderating effect analysis, variables were centered to reduce multicollinearity before constructing the interaction term (work-family conflict × sense of coherence); hierarchical regression was conducted to test the significance of the interaction term, and simple slope analysis was further performed to interpret the moderating effect by plotting slopes at ±1 standard deviation (SD) of sense of coherence (low vs. high levels). All tests were two-tailed, and a P-value < 0.05 was considered statistically significant.

## 3 Results

This cross-sectional study included a survey of 527 medical staff. As presented in **Table 1**, the majority of participants were female (75.9%), and nearly half (49.5%) had attained an educational level of junior college or below. Most respondents held junior-level positions (79.1%), were unmarried (72.1%), and had no children (74.4%). The sample consisted of doctors, nurses, and medical technicians in comparable proportions (35.3%, 32.3%, and 32.4%, respectively). The mean age was 26.5 ± 7.7 years, indicating a relatively young workforce. The overall sense of coherence (SOC) score was 49.7 ± 7.4, which was below the midpoint of the scale. Work-family conflict was generally low (26.3 ± 8.3), with time-based conflict being the most pronounced dimension (51.6 ± 15.0). The average score for workplace ostracism was 16.2 ± 5.7.

**Table 1:** Demographic and mental health information of the surveyed healthcare workers.

Variables	N(%) or M±SD
Age (years)	26.5±7.7
Gender	
Male	127(24.1)
Female	400(75.9)
Education level	
Junior college or below	261(49.5)
Bachelor	107(20.3)
Master	151(28.7)
Doctor	8(1.5)
Professional title	
Junior	417(79.1)
Intermediate	78(14.8)
Associate Senior	20(3.8)
Senior	12(2.3)
Marital status	
Unmarried	380(72.1)
Married	147(27.9)
Position	
Doctor	186(35.3)
Nurse	170(32.3)
Medical technician	171(32.4)
Child stage	
No children	392(74.4)
Infancy	54(10.3)
Preschool age	27(5.1)
School age	24(4.6)
Adolescence or above	30(5.7)
Night shifts per week	
1	121(23.0)
2	170(32.3)
3	187(35.5)
4	49(9.3)
Sense of coherence (points)	49.7±7.4
Comprehensibility	19.3±3.6
Manageability	19.0±3.8
Meaningfulness	15.5±2.6
Work-family conflict (points)	26.3±8.3
Time conflict	51.6±15.0
Strain conflict	18.1±5.4
Behavioral conflict	17.4±5.3
Workplace ostracism (points)	16.2±5.7

The results of multiple linear regression analyses revealed several significant predictors (see **Table 2**). Each additional year of age was positively associated with higher SOC scores (*β* = 0.126, 95% CI: 0.045 to 0.208, *P* = 0.002). Male healthcare workers reported significantly higher levels of workplace ostracism (*β* = 2.216, 95% CI: 0.556 to 3.878, *P* = 0.009) and work-family conflict (*β* = 5.494, 95% CI: 2.531 to 8.458, *P* < 0.001) compared to their female counterparts. Participants with a bachelor’s degree exhibited significantly higher levels of workplace ostracism (*β* = 2.345, 95% CI: 0.472 to 4.220, *P* = 0.014), work-family conflict (*β* = 3.808, 95% CI: 0.473 to 7.145, *P* = 0.025), and SOC (*β* = 2.452, 95% CI: 0.796 to 4.110, *P* = 0.004) than those with a junior college degree or below. Individuals holding a master’s degree demonstrated significantly higher work-family conflict (*β* = 5.972, 95% CI: 3.000 to 8.944, *P* < 0.001). Possessing an intermediate professional title was associated with higher SOC scores compared to junior titles (*β* = 2.482, 95% CI: 0.700 to 4.264, *P* = 0.006), and married participants reported significantly higher SOC than unmarried respondents (*β* = 1.863, 95% CI: 0.460 to 3.268, *P* = 0.009). Having a preschool-aged child was associated with increased work-family conflict (*β* = 6.074, 95% CI: 0.239 to 11.909, *P* = 0.041), whereas having a child in adolescence or older was linked to higher SOC (*β* = 3.555, 95% CI: 0.816 to 6.295, *P* = 0.011). Medical technicians experienced significantly lower workplace ostracism than doctors (*β* = –2.664, 95% CI: –4.388 to –0.940, *P* = 0.003). Each additional night shift per week was associated with increased workplace ostracism (*β* = 0.884, 95% CI: 0.117 to 1.652, *P* = 0.024) and work-family conflict (*β* = 2.188, 95% CI: 0.816 to 3.560, *P* = 0.002), as well as decreased SOC (*β* = –0.681, 95% CI: –1.362 to –0.004, *P* = 0.005).

**Table 2:** Linear regression analysis of each variable with workplace ostracism, work-family conflict, and sense of coherence.

Variables	Workplace ostracism		Work-family conflict		Sense of coherence	
	*β*(95%CI)	*P-value*	*β*(95%CI)	*P-value*	*β*(95%CI)	*P-value*
Age	0.086 (−0.006, 0.179)	0.068	0.108 (−0.058, 0.275)	0.200	0.126 (0.045, 0.208)	0.002
Gender						
Female	Reference		Reference		Reference	
Male	2.216 (0.556,3.878)	0.009	5.494 (2.531, 8.458)	<0.001	−0.904 (−2.383, 0.576)	0.231
Education level						
Junior College or Below	Reference		Reference		Reference	
Bachelor	2.345 (0.472, 4.220)	0.014	3.808 (0.473, 7.145)	0.025	2.452 (0.796, 4.110)	0.004
Master	1.596 (−0.072, 3.266)	0.061	5.972 (3.000, 8.944)	<0.001	0.391 (−1.085, 1.867)	0.603
Docter	−1.008 (−6.868, 4.852)	0.736	1.142 (−9.288, 11.574)	0.830	−2.520 (−7.702, 2.660)	0.340
Professional title						
Junior Position	Reference		Reference		Reference	
Intermediate	1.244 (−0.781, 3.270)	0.228	0.748 (−2.888, 4.385)	0.686	2.482 (0.700, 4.264)	0.006
Associate Senior	−0.038 (−3.798, 3.720)	0.984	−1.443 (−8.191, 5.304)	0.674	2.376 (−0.930, 5.684)	0.159
Senior	1.327 (−3.480, 6.136)	0.588	4.806 (−3.825, 13.437)	0.274	1.610 (−2.620, 5.840)	0.455
Marital status						
Unmarried	Reference		Reference		Reference	
Married	1.166 (−0.425, 2.758)	0.150	2.325 (−0.530, 5.180)	0.110	1.863 (0.460, 3.268)	0.009
Child stage						
No Children	Reference		Reference		Reference	
Infancy	0.496 (−1.090, 7.423)	0.682	3.166 (−1.090, 7.423)	0.144	0.814 (−1.285, 2.914)	0.446
Preschool age	2.015 (0.239, 11.909)	0.226	6.074 (0.239, 11.909)	0.041	0.647 (−2.230, 3.525)	0.658
School age	1.223 (−3.250, 9.083)	0.486	2.916 (3.250, 9.083)	0.353	2.638 (−0.402, 5.680)	0.089
Adolescence or above	1.881 (−8.355, 2.755)	0.235	−2.800 (−8.355, 2.755)	0.323	3.555 (0.816, 6.295)	0.011
Position						
Doctor	Reference		Reference		Reference	
Nurse	−0.402 (−2.128, 1.325)	0.648	−2.672 (−5.758, 0.414)	0.090	−0.281 (−1.825, 1.264)	0.721
Medical technician	−2.664 (−4.388, −0.940)	0.003	−6.021 (−9.103, −2.940)	<0.001	0.120 (−1.422, 1.662)	0.878
Night shifts per week	0.884 (0.117, 1.652)	0.024	2.188 (0.816, 3.560)	0.002	−0.681 (−1.362, −0.004)	0.005

**Table 3** presents the correlation analysis among work-family conflict, sense of coherence, and workplace ostracism. The correlation coefficient between work-family conflict and workplace ostracism is 0.613, indicating a strong positive correlation; thus, higher levels of work-family conflict are associated with more severe workplace ostracism. Conversely, the correlation coefficient between work-family conflict and SOC is −0.163, indicating a negative correlation. The findings indicate that an increase in work-family conflict is associated with a decrease in SOC. The correlation coefficient between SOC and workplace ostracism is −0.212, suggesting a significant negative correlation. This implies that a lower SOC corresponds to a higher degree of workplace ostracism.

**Table 3:** Correlation analysis of work-family conflict, sense of coherence and workplace ostracism.

	Work-family conflict	Sense of coherence	Workplace ostracism
Work-family conflict	1.000*		
Sense of coherence	−0.163*	1.000*	
Workplace ostracism	0.613*	−0.212*	1.000*

*P-value<0.05.

**Figure 1:**
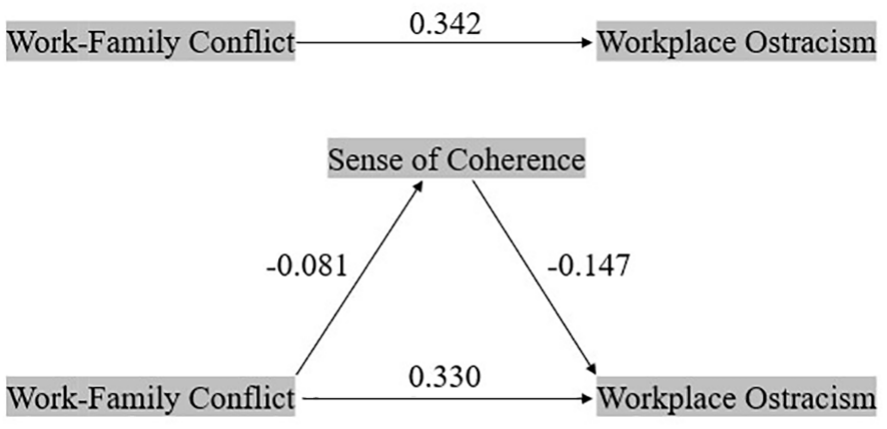
Model for evaluating the moderated mediation effect of sense of coherence between work-family conflict and workplace ostracism.

The mediation effect analysis, detailed in **Table 4**, reveals that prior to adjusting for confounding variables, the interaction between work-family conflict and SOC significantly predicted workplace ostracism (*β* = 0.331, *P* < 0.001), accounting for 3.0% of the total effect. Even after controlling for variables such as gender, age, education level, marital status, professional title, child stage, position, and average number of night shifts per week, the interaction between work-family conflict and SOC continued to significantly predict workplace ostracism (*β* = 0.330, *P* < 0.001), with the mediation effect comprising 3.6% of the total effect. The 95% confidence interval for the Bootstrap test results of the mediation model, after controlling for confounding variables, excluded 0, indicating a significant mediation effect. Consequently, SOC serves as a partial mediator in the pathway through which work-family conflict influences workplace ostracism. **Figure 1** illustrates the mediation effect path diagram after adjusting for confounding factors.

**Table 4:** Mediation effect analysis of sense of coherence between work-family conflict and workplace ostracism.

	Before adjusting for confounding variables		After adjusting for confounding variables ^#^		Bootstrap 95%CI^#^	
	*β*	*P-value*	*β*	*P-value*	Lower	Upper
Total effect	0.342	<0.001	0.342	<0.001	0.286	0.397
Indirect effect	0.010	0.013	0.012	0.009	0.001	0.023
Direct effect	0.331	<0.001	0.330	<0.001	0.272	0.387
Proportion of effect	3.0%		3.6%			

^#^Adjusted confounding variables: gender, age, education level, marital status, professional title, child stage, position, night shifts per week.

**Figure 2:**
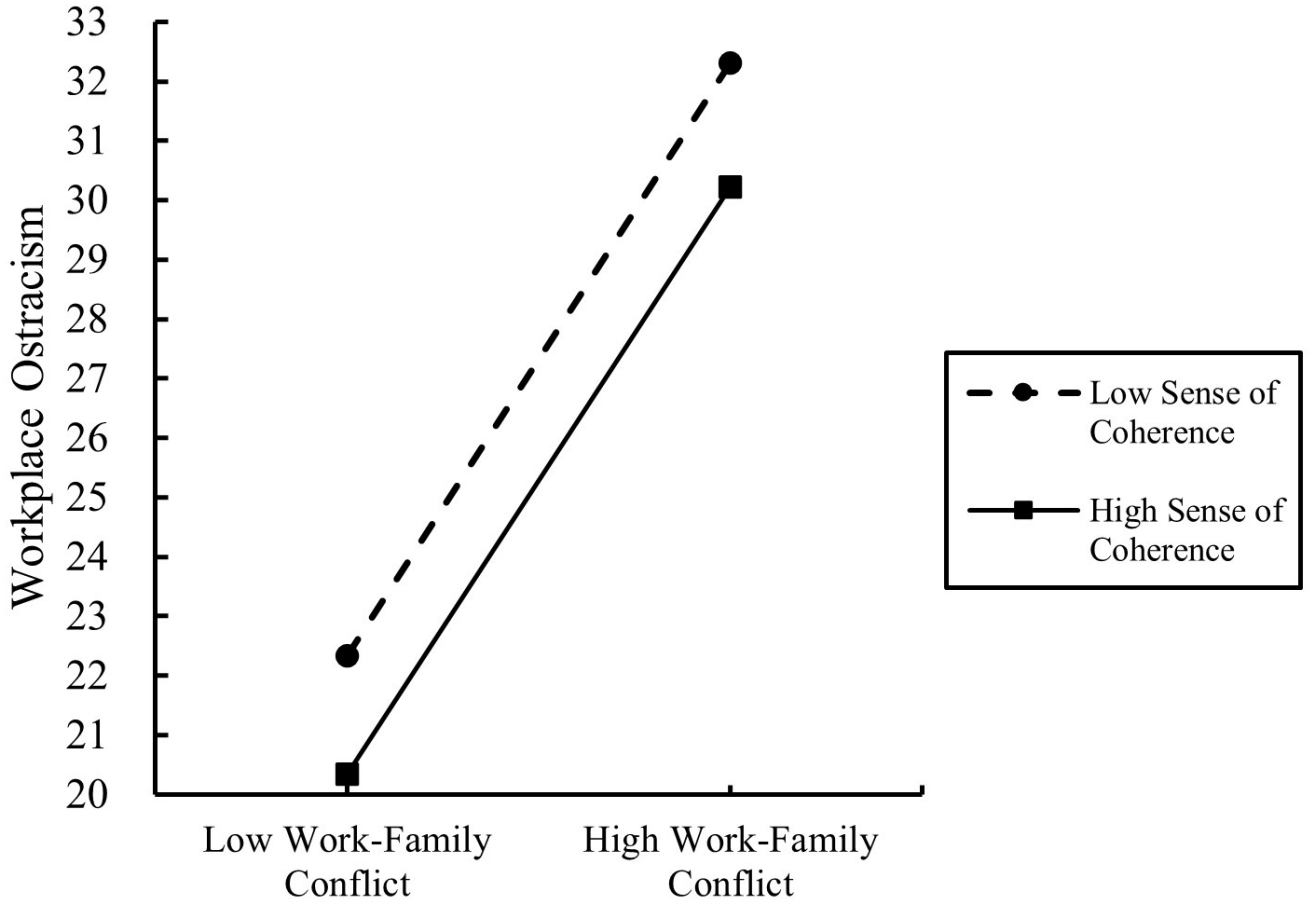
The moderating role of sense of coherence in the relationship between work-family conflict and workplace ostracism.

To further explore the moderating effect of SOC, a simple slope test was conducted in this study, and the results are presented in **Figure 2**. Upon controlling for variables such as gender, age, education level, marital status, professional title, child stage, position, and average number of night shifts per week, it was found that work-family conflict exerted a stronger positive predictive effect on workplace ostracism among healthcare workers with a low SOC (M − 1SD) (*simple slope* = 0.333, *P* < 0.001) compared to those with a high SOC (M + 1SD) (*simple slope* = 0.330, *P* < 0.001). These findings suggest that the relationship between work-family conflict and workplace ostracism is moderated by SOC, with the effect being more pronounced among medical and healthcare professionals with a low SOC.

## 4 Discussion

### 4.1 Influences of demographic and occupational characteristics on core variables

Several key findings emerge regarding the general characteristics and circumstances of medical staff based on the study results. As individuals age, advance in professional titles, and are married, their sense of coherence (SOC) scores gradually increase, reflecting a strengthened capacity to cope with diverse pressures and stressors. Men are more susceptible to workplace ostracism and work-family conflict than women. Healthcare workers with higher educational attainment demonstrate distinct patterns in workplace ostracism, work-family conflict, and SOC: while they are more prone to experiencing workplace ostracism and work-family conflict, they tend to hold more positive overall perceptions and attitudes toward work and life.

Work-family conflict markedly increases when the youngest child is in the preschool stage; conversely, SOC is significantly enhanced when the youngest child enters adolescence or beyond. This indicates that changes in children’s developmental stages can shape work-family conflict and SOC among healthcare workers. Compared to doctors, medical technicians demonstrate lower levels of workplace ostracism and SOC—though they experience less ostracism, their psychological resilience and overall perceptions and attitudes toward work and life are less favorable. Each additional weekly night shift exacerbates both workplace ostracism and work-family conflict while reducing SOC, highlighting that night shift frequency exerts a notable impact on healthcare workers’ perceptions and experiences related to work and life.

### 4.2 Dimension characteristics of work-family conflict and overall SOC level

Among work-family conflict dimensions, time conflict yielded the highest scores, which were significantly higher than those of strain conflict and behavioral conflict. This may stem from the fact that most respondents are in the early stages of their careers, characterized by heavy workloads and the necessity of adapting to new workplace environments. Compared with senior medical staff, these individuals have limitations in establishing social relationships and utilizing resources (22), which further amplifies work-related stress.

Additionally, many respondents have young children requiring substantial parental time and effort for care, rendering it difficult to balance time allocation between professional and familial responsibilities and thereby increasing conflict risk. Another notable finding is that the majority of respondents had SOC scores below 63, indicating a relatively low overall level of SOC. This may be attributed to medical staff’s prolonged exposure to heavy workloads and extended working hours, which leads to inadequate psychological resilience and subsequently contributes to issues such as job burnout, anxiety, depression, and work-family conflict (9, 18). These challenges ultimately impact their satisfaction with both work and personal life, resulting in lower SOC scores.

### 4.3 Predictive effect of work-family conflict on workplace ostracism

The study found that work-family conflict significantly and positively predicts workplace ostracism—specifically, the more severe the work-family conflict, the more pronounced an individual’s perception of workplace ostracism. Work and family are two core domains of daily life. According to role theory, conflicting demands from multiple simultaneous roles can trigger the depletion of psychological resources, including negative emotions and maladaptive cognitive tendencies (23).

Influenced by traditional Chinese culture, which emphasizes family care and dedication, individuals experiencing work-family conflict may develop negative attitudes toward work or experience emotional exhaustion (24), which adversely affects their job performance and ultimately contributes to workplace ostracism.

### 4.4 Mediating role of SOC between work-family conflict and workplace ostracism

This study innovatively identifies that SOC mediates the relationship between work-family conflict and workplace ostracism, meaning work-family conflict can indirectly influence workplace ostracism through SOC. Based on Conservation of Resources (COR) theory, individuals prioritize resource preservation over acquisition and exhibit heightened sensitivity to resource depletion (25). Thus, when individuals perceive resource loss (e.g., psychological overload resulting from competing demands of work and family roles), they may adopt strategies to mitigate such losses, such as adjusting work attitudes or reducing proactive behaviors (26).

It can be hypothesized that subsequent gradual increases in pressure may cause employees to become increasingly exhausted following repeated experiences of ostracism (11), leading to increased dissatisfaction and conflict that further exacerbates workplace ostracism. According to the ABC theory of emotions, emotional distress is not directly caused by stressors but rather depends more on an individual’s cognitive evaluation of such stressors (27). When facing negative events and adverse emotions, a strong SOC helps individuals resist negative impacts, facilitate effective coping strategies, and activate internal protective resources, thereby enhancing positive psychological states (28). Therefore, in the pathway where work-family conflict leads to workplace ostracism, higher SOC levels can better regulate psychological states, alleviate negative impacts on work performance, and reduce the likelihood of workplace ostracism. These findings have been relatively underexplored in prior research.

### 4.5 Study strengths

This study offers several notable strengths. Firstly, prior research on workplace ostracism and SOC in healthcare has predominantly focused on nurses. In contrast, the present study includes a broader range of occupational groups, including doctors and medical technicians. Secondly, it examines the influence of children’s developmental stages on healthcare professionals’ psychological well-being—a factor rarely addressed in previous studies. Lastly, to ensure data diversity and validity, we collected data from one tertiary hospital in each of three distinct regions in China (Northwest, Northeast, and South).

### 4.6 Study limitations

Despite these findings and strengths, the study has several limitations. Firstly, due to limitations in the questionnaire distribution method, the surveyed population was predominantly composed of younger and lower-ranking medical staff. Thus, the conclusions drawn are more applicable to healthcare workers with shorter tenure and lower professional titles. Future research should include a more diverse sample, including older and higher-ranking medical staff, to enhance sample representativeness and strengthen finding persuasiveness.

Secondly, the study was conducted within the Chinese cultural context, so caution is warranted when extrapolating findings to countries with different cultural backgrounds. Thirdly, the cross-sectional design assesses relationships among variables at a single time point without longitudinal follow-up. Consequently, several measured variables may change over time, which could affect the scores of assessed indicators. Finally, this study focuses exclusively on currently employed individuals and does not include those who may have left their positions due to workplace ostracism (29).

### 4.7 Practical implications

Medical professionals frequently face mental health challenges, yet there is a notable lack of guidance on identifying and supporting those severely affected by such issues (30). The impact of mental health on clinical practice remains a persistent concern for many healthcare providers. While ensuring patient safety, it is also imperative to prioritize the mental well-being of medical staff.

Healthcare institutions should strive to create more supportive work environments, improve employee well-being, and closely monitor their mental health. By fostering stronger SOC, alleviating work-family conflicts, and reducing perceptions of workplace ostracism, not only can professional satisfaction be enhanced, but medical professionals can also be more fully engaged in advancing healthcare.

## 5 Conclusions

Building on the key findings presented in the discussion, this study confirms that age, gender, professional title, marital status, children’s developmental stage, occupation, and average weekly night shift frequency are significant factors influencing levels of work-family conflict, workplace ostracism, and SOC among medical staff. Specifically, work-family conflict significantly and positively predicts workplace ostracism among this group, and SOC plays a mediating role in this relationship—with the mediating effect being more pronounced among medical staff with lower SOC. These results collectively highlight the need for medical institutions to implement targeted measures to safeguard the mental health of medical staff, particularly by addressing the impacts of work-family conflict, workplace ostracism, and SOC.

## Data Availability

The original contributions presented in the study are included in the article/**Supplementary Material**. Further inquiries can be directed to the corresponding author.
