# Supplementary material for: A mediating effect of sense of coherence on the association between work-family conflict and workplace ostracism among medical staff
Source: Front Psychiatry. 2026 Feb 2;16:1584004. doi: 10.3389/fpsyt.2025.1584004 (PMC12907823; doi:10.3389/fpsyt.2025.1584004)
Supplement: Supplementary file 1 [file Table1.docx]

您好！现邀请您参加一项研究，题目是“探讨医护群体的职场排斥、心理一致感、工作-家庭冲突间的关系”。**纳入标准**：①医疗机构在岗工作的医生、护士、医技辅助人员；②年龄在18至60周岁。**排除标准**：①离退休人员；②正在休假（大于6个月）人员。

如果您符合上述条件且自愿参加此研究，请您配合完成以下调查问卷。本问卷为**匿名**填写，研究结果将以经统计分析后的数据形式发表，**不包含任何可识别的参加者信息**，以保护您的隐私。请您如实填写，选择最符合您本人情况的选项。**所有问题均为单项选择**，切勿多选。

感谢您的配合！

Dear Participant,

You are invited to participate in a research study entitled: **"Exploring the Relationships Among Workplace Ostracism, Sense of Coherence, and Work-Family Conflict in Healthcare Professionals"**.

**Eligibility Criteria**

- **Inclusion**: Currently employed physicians, nurses, or allied health professionals in medical institutions; Aged 18 to 60 years.
- **Exclusion**: Retired healthcare workers; Individuals currently on leave (>6 months).

If you meet the above criteria and **voluntarily agree to participate**, please complete the following questionnaire.

**Confidentiality Assurance**

- All responses will be **collected anonymously**
- Research findings will be published **exclusively as statistically analyzed aggregated data**
- **No personally identifiable information** will be disclosed
- Your privacy will be **strictly maintained**

**Instructions**

- Provide truthful responses reflecting your actual situation
- Select the **single most appropriate option** for each item
- **Do not select multiple answers** for any question

We sincerely appreciate your time and contribution to this study.

**一般人口统计信息调查问卷**

**General Demographic Information Questionnaire**

注意：所有问题均为单项选择，切勿多选。

1. 您的性别：

□女性 □男性

2. 您的年龄是______周岁

3. 您的教育程度：

□大专及以下 □本科 □硕士 □博士

4. 您的职称：

□初级 □中级 □副高级 □正高级

5. 您的婚姻状况：

□未婚 □已婚 □离婚 □丧偶

6. 您的孩子阶段（如有多个孩子，以年龄最小者为准）：

□无孩子 □婴幼儿 □学龄前期 □学龄期 □青春期及以上

7. 您的工作岗位：

□医生 □护士 □医技辅助

8. 您平均每周夜班的数量：

□1 □2 □3 □4

**工作-家庭冲突量表**

**Work-family Conflict Scale**

注意：所有问题均为单项选择，切勿多选。

1. 我的工作使我无法参与家庭活动

□非常不同意 □不太同意 □不确定 □比较同意 □非常同意

2. 我花在工作上的时间太多，这就使得我没有足够的时间参与家庭活动

□非常不同意 □不太同意 □不确定 □比较同意 □非常同意

3. 我不得不牺牲家庭活动的时间来完成必须的工作任务

□非常不同意 □不太同意 □不确定 □比较同意 □非常同意

4. 我花在家庭义务上的时间通常会干涉到我工作任务的完成

□非常不同意 □不太同意 □不确定 □比较同意 □非常同意

5. 由于要陪家人，这使得我不能参加那些对我职业生涯有益的工作活动

□非常不同意 □不太同意 □不确定 □比较同意 □非常同意

6. 我不得不牺牲完成工作任务的时间来完成家庭义务

□非常不同意 □不太同意 □不确定 □比较同意 □非常同意

7. 当我工作完回到家，我通常会感到太累而不想参加家庭活动

□非常不同意 □不太同意 □不确定 □比较同意 □非常同意

8. 当我工作完回到家，我通常会感到情绪枯竭而无法履行家庭义务

□非常不同意 □不太同意 □不确定 □比较同意 □非常同意

9. 由于工作上的事情，有时当我回到家也会感到有压力

□非常不同意 □不太同意 □不确定 □比较同意 □非常同意

10. 我通常会在工作中还一心想着家里的事

□非常不同意 □不太同意 □不确定 □比较同意 □非常同意

11. 由于家庭责任给我带来的压力，我很难专心于我的工作

□非常不同意 □不太同意 □不确定 □比较同意 □非常同意

12. 家庭生活带来的紧张和焦虑削弱了我工作上的能力

□非常不同意 □不太同意 □不确定 □比较同意 □非常同意

13. 我有效解决工作问题的行为并不能让我很好

□非常不同意 □不太同意 □不确定 □比较同意 □非常同意

14. 工作上的那些必要和有效的行为对家庭反而是无益的

□非常不同意 □不太同意 □不确定 □比较同意 □非常同意

15. 我在工作上的那些出色行为并不能帮助我成为更好的家长和伴侣

□非常不同意 □不太同意 □不确定 □比较同意 □非常同意

16. 我有效解决家庭中问题的行为并不能让我很好的解决工作上的问题

□非常不同意 □不太同意 □不确定 □比较同意 □非常同意

17. 家庭中那些必要和有效的行为对工作反而是无益的

□非常不同意 □不太同意 □不确定 □比较同意 □非常同意

18. 那些有益于解决家庭中问题的行为似乎不能有效的作用于工作

□非常不同意 □不太同意 □不确定 □比较同意 □非常同意

**职场排斥量表**

**Workplace Ostracism Scale**

注意：所有问题均为单项选择，切勿多选。

1. 在以往的工作中，有的同事会忽视我的感受或观点

□完全不符合 □不太符合 □不确定 □比较符合 □完全符合

2. 当我向其他同事问好时，有时会得不到回应

□完全不符合 □不太符合 □不确定 □比较符合 □完全符合

3. 在工作中，有的同事曾经因我靠近某个区域而选择离开

□完全不符合 □不太符合 □不确定 □比较符合 □完全符合

4. 在职工餐厅，我有时会独自吃工作餐

□完全不符合 □不太符合 □不确定 □比较符合 □完全符合

5. 有的同事曾经不愿意与我一起协作共事

□完全不符合 □不太符合 □不确定 □比较符合 □完全符合

6. 在工作中，我发现同事有时不会关注到我

□完全不符合 □不太符合 □不确定 □比较符合 □完全符合

7. 同事有时会不愿让我加入他们的交谈

□完全不符合 □不太符合 □不确定 □比较符合 □完全符合

8. 同事有时会不愿和我说话

□完全不符合 □不太符合 □不确定 □比较符合 □完全符合

9. 在工作中，有时同事会忽视我的存在

□完全不符合 □不太符合 □不确定 □比较符合 □完全符合

10. 在休息时间，同事外出时，没有邀我同去或问我是否需要带点什么

□完全不符合 □不太符合 □不确定 □比较符合 □完全符合

**心理一致感量表**

**Sense of Coherence Scale**

注意：所有问题均为单项选择，切勿多选。

1. 你是不是常常觉得自己对周围发生的事并不关心？

□从来没有 □几乎没有 □较少 □说不清 □发生过 □较多 □经常

2. 你本来以为很了解的人做出让你吃惊的行为，这种情况在过去是不是经常发生？

□从来没有 □几乎没有 □较少 □说不清 □发生过 □较多 □经常

3. 你指望的人却让你失望，这种情况

□从来没有 □几乎没有 □较少 □说不清 □发生过 □较多 □经常

4. 你是不是经常感到自己受到不公正的对待？

□经常 □较多 □偶尔 □说不清 □较少 □几乎没有 □从来没有

5. 你是不是经常感到自己处于陌生的、不知如何是好的环境中？

□经常 □较多 □偶尔 □说不清 □较少 □几乎没有 □从来没有

6. 你是否经常有非常复杂的、混合的感情和念头？

□非常频繁 □经常 □偶尔 □说不清 □较少 □几乎没有 □从来没有

7. 你是不是经常产生自己不愿产生的情绪？

□非常频繁 □经常 □偶尔 □说不清 □较少 □几乎没有 □从来没有

8. 很多人，哪怕是很有天分的人，有时在一定环境下也会感到很失败的。在过去的经历中，你是否常有这种失败的感受？

□从来没有 □几乎没有 □较少 □说不清 □发生过 □较多 □经常

9. 每天做的这些事没什么意义，你产生这种想法的频率？

□非常频繁 □经常 □偶尔 □说不清 □较少 □几乎没有 □从来没有

10. 你是不是常有失控的感觉？

□非常频繁 □经常 □偶尔 □说不清 □较少 □几乎没有 □从来没有

11. 到目前为止，你的生活

□根本没有目标 □几乎没有目标 □不太有目标 □说不清 □有目标

□生活目标比较明确 □生活目标非常明确

12. 当遇到问题或事情，您发现自己一般都会

□低估或高估了它的重要性 □很难估计准 □估计不太准 □说不清

□比较准确评价这件事 □准确评价这件事 □非常正确评价这件事

13. 做那些你每天都做的事对于你来说

□是极大的快乐和满足 □比较快乐和满足 □有点快乐 □说不清

□有点不快乐 □很不快乐 □是痛苦和烦恼的源泉
